# Clinical Features and Outcomes of Dipeptidyl Peptidase-4 Inhibitor-Associated Bullous Pemphigoid (DPP4i-Associated BP) in Thai Patients

**DOI:** 10.1155/2020/8832643

**Published:** 2020-10-10

**Authors:** Yotsapon Thewjitcharoen, Ekgaluck Wanothayaroj, Chattip Thammawiwat, Sriurai Porramatikul, Chuleekorn Vorayingyong, Soontaree Nakasatien, Sirinate Krittiyawong, Kumutnart Chanprapaph, Thep Himathongkam

**Affiliations:** ^1^Diabetes and Thyroid Center, Theptarin Hospital, Bangkok, Thailand; ^2^Department of Dermatology, Theptarin Hospital, Bangkok, Thailand; ^3^Division of Dermatology, Department of Medicine, Ramathibodi Hospital, Bangkok, Thailand

## Abstract

The use of dipeptidyl peptidase-4 inhibitors (DPP4i) appears to be associated with a small but significantly elevated risk of bullous pemphigoid (BP). Although the pathogenic mechanism of DPP4i-associated BP remains unclear, this adverse event is reported with multiple gliptins, suggesting a class effect. However, previous studies from various countries showed that vildagliptin had been implicated in most cases. The aim of this study was to illustrate a case series of DPP4i-associated BP in Thai patients. We conducted a retrospective study from consecutive cases of BP in people with type 2 diabetes mellitus (T2DM) from January 2008, the year in which the first DPP4i was introduced in Thailand, until December 2019. During the study period, 10 BP patients with T2DM were identified. A total of 5 DPP4i-associated BP (3 on vildagliptin, 1 on linagliptin, and 1 on sitagliptin) were found. All patients were male with a mean age at BP development of 80.4 years (73–86 years). All patients had a long-standing duration of diabetes (median duration 34 years), and mean A1C was 7.5 ± 1.4%. The median time to BP development after the introduction of DPP4i was 64 months (range 20–128 months). The severity of BP was classified as mild in 2 cases. In all cases, the association between the drug intake and BP onset was classified as “possible” according to the Naranjo causality scale. All of the patients continued taking DPP4i after BP diagnosis, and one patient died of lung cancer 18 months after BP diagnosis. Only 2 patients could achieve complete remission at least 2 months after stopping DPP4i. Our case series demonstrated a potential link between DPP4i and the development of BP, which mainly occurred in very elderly male patients. The latency period from an introduction of DPP-4i could be several years, and the clinical course after DPP4i discontinuation varied. Clinicians prescribing DPP4i should be aware of this association and consider stopping this medication before a refractory disease course ensues.

## 1. Introduction

Bullous pemphigoid (BP) is an uncommon acquired autoimmune disease that occurs mainly in elderly patients with multiple comorbidities and is a relatively rare condition (21–66 cases per a million people in European studies) [1]. However, the incidence of BP is increasing, and recent several studies confirmed that dipeptidyl peptidase-4 inhibitors (DPP4i), which is a common class of antidiabetic medication among elderly patients due to its safety profiles, had been associated with BP [2–5]. A group of Japanese researchers suggested that DPP4i-associated BP displays unique clinical and immunological features with a predominate of the noninflammatory phenotype, and autoantibodies target distinct epitopes on BP180 [6]. However, these findings need to be confirmed in other ethnicities.

Available data from the pharmacovigilance database and cohort studies worldwide had emerged to reveal that various DPP4is, especially vildagliptin, are a potential trigger agent for the development of BP [7–11]. However, most case series on DPP4i-associated BP had been reported from Japan and with subsequently large database from Western countries illustrating the same potential association. More accumulated case series in other ethnicities are required to better understand the clinical features and outcomes of this rare adverse event from a commonly used antidiabetic medication for people with type 2 diabetes mellitus (T2DM). The aim of this study was to illustrate a case series of DPP4i-associated BP in Thai patients from Theptarin Hospital, one of the largest diabetes centers in Bangkok. The clinical outcomes were also reported after discontinuation of DPP4i.

## 2. Materials and Methods

We conducted a retrospective study from consecutive cases of BP in people with T2DM from January 2008, the year in which the first DPP4i was introduced in Thailand, to December 2019 to assess whether the use of DPP4i in T2DM is potentially related to the occurence of BP. The clinical characteristics, latency period, Naranjo adverse drug reaction probability scale, and clinical outcomes were evaluated. The Naranjo scale is a validated rating scale to determine the likelihood of whether an adverse event is actually due to the drug rather than a result of other factors [12]. This scoring system is composed of 10 questions with 3 possible answers (yes/no/do not know) and 4 adverse events categories (doubtful, possible, probable, and definite).

The diagnosis of BP was based on typical or consistent clinical features, compatible histological findings, and/or positive immunopathological studies, including direct and indirect immunofluorescence microscopy and enzyme-linked immunosorbent assays (ELISA) for BP180 and BP230. BP180 is a transmembrane glycoprotein, which connects intracellular (BP230) and extracellular hemidesmosome proteins. Due to the high specificity of both tests, serum anti-BP180 IgG and anti-BP230 IgG have been used in establishing the diagnosis of BP [13]. We also assessed BP severity by determining whether steroids were administered systemically and whether patients were hospitalized. After the withdrawal of DPP4i, complete remission was defined as no treatment or receiving minimal therapy for at least 2 months [14]. Minimal therapy is defined as less than or equal to 0.1 mg/kg/day of prednisone or 20 g/week of topical clobetasol propionate. This study was approved by the Institutional Review Board committee of Theptarin Hospital (EC No.6–2019).

## 3. Results

**Figure 1:**
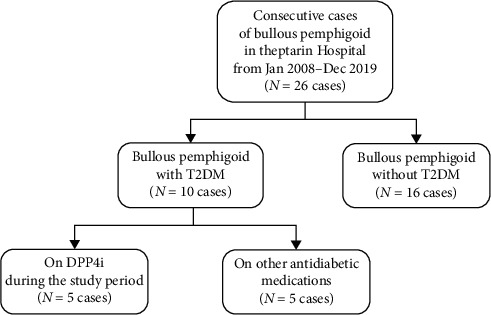
Flow selection of studied patients.

Between January 2008 and December 2019, a total of 26 BP patients were identified. Among these, 10 patients had T2DM and 5 of whom were under DPP4i treatment (3 on vildagliptin, 1 on linagliptin, and 1 on sitagliptin) as shown in Figure 1. During this period, a total of 3,678 T2DM were first prescribed with various DPP4is and were followed up regularly at our hospital. Therefore, the estimated incidence of DPP4i-associated BP was 1.4 cases per 1,000 DPP4i-treated patients. All patients were male, and the mean age at BP development was 80.4 years (73–86 years). All patients had a long-standing duration of diabetes (median duration of 32 years), and mean A1C was 7.5 ± 1.4%. The median time to BP development after the introduction of DPP4i was 64 months (range 20–128 months). The severity of BP was classified as mild in 2 cases. In all cases, the association between the drug intake and BP onset was classified as “possible” according to the Naranjo causality scale. The median follow-up period after the diagnosis of BP was 32 months (range 7–35 months).

All of the patients continued taking DPP4i after BP diagnosis. DPP4i withdrawal was attempted in all cases except Case no. 1 who passed away of lung cancer at 13 months after the diagnosis of BP. The details and outcomes of each case are illustrated in the followings sections and are summarized in Table 1.

## 4. Case Reports

### 4.1. Case 1

An 87-year-old man with T2DM, hypertension, and chronic kidney disease (eGFR 55 ml/min/1.73 m^2^) presented with erythematous vesiculobullous lesions on both the legs after 40 months of treatment with glipizide, metformin, and linagliptin. His other medications included candesartan, rosuvastatin, and folic acid. Skin biopsies were taken, and the diagnosis of bullous pemphigoid was suspected. No direct immunofluorescence (DIF) method or serological tests were performed to further confirm the diagnosis. Topical treatment of clobetasol propionate 0.05% ointment was given without oral steroid. All of his medications were maintained after the diagnosis of bullous pemphigoid. Skin lesions were partially improved after topical steroid treatment only. At 6 months later, he had been diagnosed with nonsmall cell lung carcinoma accidentally from routine chest X-ray imaging. The patient had been under palliative treatments for 12 months, until he passed away from obstructive pneumonia.

### 4.2. Case 2

A 74-year-old man with poorly controlled T2DM, hypertension, dyslipidemia, ischemic heart disease, heart failure, and predialysis end stage renal disease presented with erythematous bullous lesions in the back in December 2017 and then progressed to lower extremities within 3 months. He had been treated with several DPP4i in the past 10 years, starting from sitagliptin in 2008–2014, linagliptin 2014–2016, and finally vildagliptin since October 2016 (15 months before eruption of skin lesions). Other medications included metformin, pioglitazone, empagliflozin, valsartan, spironolactone, simvastatin, ezetimibe, and clopidogrel. Skin biopsies confirmed the diagnosis of bullous pemphigoid with the positive DIF method. Topical treatment of clobetasol propionate 0.05% ointment and oral prednisolone 20 mg per day were given. No mucosal involvement was found. All of his medications were maintained after the diagnosis of bullous pemphigoid. Skin lesions were improved after oral prednisolone and then gradually tapered to 5 mg per day within 6 months. Vildagliptin was discontinued in July 2018 due to progression of renal failure requiring hemodialysis. Current, his BP lesions are stable with oral prednisolone 5 mg per day, and his glycemic control is treated with multiple daily insulin injections. In summary, the clinical course of BP was easily controlled after stopping vildagliptin; however, remission could not be achieved.

### 4.3. Case 3

An 85-year-old man with T2DM and dyslipidemia presented with gradual eruption of erythematous vesiculobullous lesions at his forearms and then spread to all over the body within 1 month. No mucosal involvement was found. The diagnosis of severe bullous pemphigoid was confirmed with typical histological findings, positive DIF, and positive serum anti-BP180 and anti-BP230. He had been hospitalized and treated with intravenous steroid and oral azathioprine 150 mg/day. His medications included glicazide, metformin, vildagliptin, and simvastatin. Vildagliptin was started for 64 months before the diagnosis of BP and was still continued for 24 months after the diagnosis. After the potential association between DPP4i and bullous pemphigoid was widely reported in 2018, vildagliptin was discontinued in October 2018. His BP maintenance treatment with oral prednisolone could be tapered from 15 mg to 2.5 mg per day within 2 months after stopping vildagliptin. Oral azathioprine also was stopped without further eruption of skin lesions. Unfortunately, the patient passed away from sudden cardiac arrest at the age of 88 years.

### 4.4. Case 4

**Figure 2:**
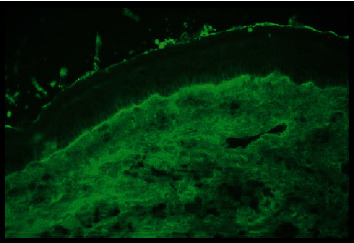
Direct immunofluorescence (DIF) analysis of skin biopsy from the patient no.4 showed a linear staining pattern with immunoglobulin G/complement C3 along the dermoepidermal junction.

**Figure 3:**
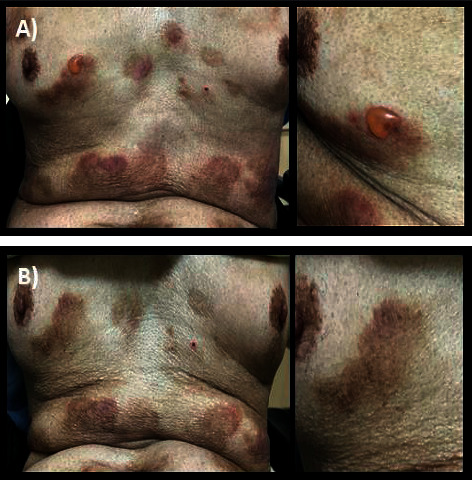
(a) Disseminated bullous pemphigoid and a close up image of bullae on the chest wall in Case no.4 before sitagliptin withdrawal and (b) improvements of skin lesions in this patient after 2 weeks of sitagliptin discontinuation.

An 84-year-old man with underlying T2DM, dyslipidemia, ischemic heart disease, and complete remission of colon cancer presented with gradual eruption of erythematous vesiculobullous lesions on the scalp, upper extremities, and both feet for 2 weeks. No mucosal involvement was found. The diagnosis of bullous pemphigoid was confirmed with typical histological findings and positive DIF. The DIF analysis showed a linear staining pattern with immunoglobulin G/complement C3 along the dermoepidermal junction as shown in Figure 2. Topical treatment of betamethasone dipropionate 0.05% ointment and oral prednisolone 10 mg per day were given to control his skin lesions. No recurrence of colon cancer had been found. His medications included metformin, pioglitazone, sitagliptin, rosuvastatin, and baby aspirin. Sitagliptin was started for 65 months before the diagnosis of BP and was continued for 15 months after the diagnosis. Until August 2018, sitagliptin was discontinued, and oral pioglitazone was titrated up to 30 mg per day due to increasingly reported cases of DPP4i-associated BP. His skin lesions went into remission without any oral steroid treatment within 2 weeks after DPP4i discontinuation. The clinical pictures before and after DPP4i withdrawal in this patient are depicted in Figure 3.

However, the patient had relapsed BP again at 4 months later, and oral prednisolone 10 mg per day was needed to restart to control skin lesions. Later on, the skin lesions are relapsing and remitting 1 to 2 times per year without requiring hospitalization. Current, his glycemic control is stable with metformin and pioglitazone, and BP could be controlled with only topical steroid ointment.

### 4.5. Case 5

A 72-year-old man with underlying T2DM, Graves' disease, and Parkinson's disease presented with acute eruption of erythematous tense bullous lesions at the left leg after 20 months of starting vildagliptin. No mucosal involvement was found. The diagnosis of bullous pemphigoid was suspected based on typical histological examination. No direct immunofluorescence (DIF) method or serological tests were performed to further confirm the diagnosis. His medications included metformin, vildagliptin, simvastatin, Tapazole, sertraline, and levodopa/benserazide. Vildagliptin was added to metformin at 20 months earlier to better glycemic control. Topical treatment of desoximetasone 0.25% ointment and oral prednisolone 20 mg per day were given to control his skin lesions. After the BP diagnosis, vildagliptin was continued for 2 months before stopping due to the potential association of DPP4i and BP. After discontinuation, his skin lesions still progressed gradually to the trunk and upper extremities. However, oral prednisolone was tapered to 10 mg per day. Unfortunately, his Parkinson's disease progressed, and he became bed ridden. Finally, he passed away from sudden cardiac arrest at home 6 months after the diagnosis of BP. In summary, the clinical course of BP did improve after stopping vildagliptin. Underlying neurological disease and long-duration exposure of DPP4i had been postulated as potential implicating factors.

## 5. Discussion

This is the first case series from Southeast Asian patients to describe the clinical features and outcomes of DPP4i-associated BP. Based on our present series from a single center institute, the incidence of DPP4i-associated BP is rare with only 1.4 cases per 1,000 DPP4i-treated patients but slightly higher than a previous report of Japanese patients (0.9 cases per 1,000 DPP4i-treated patients) [9]. The occurrence of BP is known to be common in very elderly patients (70s-80s) with neurological diseases such as Alzheimer's disease, stroke, and Parkinson's disease. As shown in our series, some patients in our series already had some underlying conditions or were taking another potential culprit drug, which had been reported in the development of BP. Previous studies showed that the increased risk of BP has been observed in patients older than 70 years, female patients, patients with T2DM, patients having dementia, and other well-known precipitating drugs such as spironolactone [1]. However, T2DM patients taking metformin or second-generation sulphonylurea had lower BP risks when compared with DPP4i [10, 11]. Clinicians prefer prescribing DPP4i as a part of antidiabetic medications in elderly T2DM patients who are prone to hypoglycemia due to its safety profiles. Therefore, DPP4i probably increases the risk of BP but does not independently induce the disease on its own. Moreover, the latency period from an introduction of DPP4i could be several years, and the clinical course after DPP4i discontinuation varied.

DPP4 (also known as CD26) is a ubiquitous cell surface glycoprotein expressed throughout the body including the skin, and its inhibition may lead to modification of the immune response [15]. Some animal studies showed that DPP4i induces infiltration of eosinophil into the skin and also change in the antigenic properties of the epidermal basement membrane [16]. Moreover, the global effects of DPP4 inhibition might affect other subtypes of DPP (DPP8 and DPP9). However, the exact pathogenesis of DPP4i-associated BP remains unknown. A recent study from Japan suggested that HLA-DQB1 *∗* 03 : 01 could be a potential biomarker for the genetic susceptibility toward noninflammatory DPP4i-associated BP among the Japanese population [17]. Further studies are required to verify this finding in other ethnicities.

Since the first anecdotal case report of DPP4i-BP cases in 2011 [18], increasing publications from various countries confirmed that DPP4i had been implicated in the onset of BP with higher odds ratios than previously known major BP-induced drugs such as furosemide, spironolactone, and captopril. Interestingly, vildagliptin had been repeatedly reported as the most increased risk of developing BP. Our present series also corroborated this observation as 3 from 5 cases had been treated with vildagliptin even though sitagliptin was the most common used DPP4i during study period. The mechanism of vildagliptin in the pathogenesis of this rare adverse event is unknown, but the molecule of this medication and relatively lesser DPP4 selectivity of vildagliptin had been proposed as a potential mechanism [7]. Earlier reports suggested that this condition tended to develop within 12 months after the introduction of DPP4i, and BP control could be achieved in the majority of cases after drug withdrawal [7, 8]. However, it should be emphasized that BP usually has a remitting-relapsing clinical course, and most previous reports had a short follow-up duration. As demonstrated in our Case no. 4, the complete remission after DPP4i withdrawal could sustain only for 4 months, and then, low-dose oral prednisolone needed to be restarted. Another possible hypothesis is that the prolonged use of DPP4i after the onset of BP in our cases might lead to a refractory disease course as suggested in a recent review [19]. Moreover, the majority of elderly T2DM patients received polypharmacy, some of which had shown an association with BP [20]. DPP4i-associated BP could be the result of interaction of DPP4i with those medications rather than by DPP4i solely as a trigger drug. Further accumulated cases are required to understand the complete clinical course of this rare side effect.

Advanced age is associated with the onset of both BP and underlying comorbidities, so clinicians should be aware of increased mortality risk in patients with BP [21]. As revealed in our case series, 3 out of 5 cases passed away within months after the diagnosis of BP. In Case no. 1, the association between malignancy and BP remained controversial, especially in Asian patients [22]. The latest population-based study from Taiwan did not demonstrate malignancy as a risk factor for BP [23]. In light of these uncertainties, DPP4i should be stopped immediately if the patient develops BP, and the clinical course after DPP4i discontinuation should be observed closely. It also should be noted that a prodromal phase of chronic eczema or urticarial eruptions could develop before blisters appear [24]. Early consultation with dermatologists in DPP4i-treated elderly T2DM patients should be done for further investigations.

Several limitations of our study should be acknowledged. First, this is a retrospective study conducted in a tertiary diabetes center, which composed of mainly elderly T2DM patients with multiple comorbidities. Second, only one case had the serological tests for anti-BP180 and anti-BP230. Detailed studies of autoantibodies and HLA genotype should be further investigated. Third, we were not able to evaluate the BP severity score in this retrospective study [25]. Further multicenter case-control studies or prospective study should be conducted to clarify the clinical outcomes of this rare adverse effect.

In conclusion, our case series is the first study in Southeast Asia to illustrate the clinical features and outcomes of DPP4i-assoicated BP. The latency period from an introduction of DPP-4i could be several years. Clinicians prescribing DPP4i should be aware of this association and consider stopping this medication before a refractory disease course ensues.

## Figures and Tables

**Table 1:** Summary of clinical characteristics and outcomes in our case series of DPP4i-associated bullous pemphigoid.

Case no.	Age/sex	Type of DPP4i	Latency period (months)	Dechallenge	Final outcomes
1	87/M	Linagliptin	40	No	Died from CA lung
2	74/M	Vildagliptin	128	Yes, after the onset of BP 8 months	Stable with oral prednisolone 5 mg/day
3	85/M	Vildagliptin	64	Yes, after the onset of BP 24 months	Complete remission for 5 months, and then, he died from sudden cardiac arrest
4	84/M	Sitagliptin	65	Yes, after the onset of BP 15 months	Complete remission for 4 months, and then, relapsed
5	72/M	Vildagliptin	20	Yes, after the onset of BP 2 months	BP gradually progressed, and then, he died from sudden cardiac arrest

## Data Availability

The data used to support the findings of this study are available from the corresponding author upon request.
